# Neurological Disease-Affected Patients, including Multiple Sclerosis, Are Poor Responders to BKPyV, a Human Polyomavirus

**DOI:** 10.1155/2022/4864950

**Published:** 2022-07-26

**Authors:** Ilaria Bononi, Elisa Mazzoni, Silvia Pietrobon, Maria Rosa Iaquinta, Andrea Caselli, Elena Torreggiani, Maura Pugliatti, Ilaria Casetta, Massimiliano Castellazzi, Enrico Granieri, Fernanda Martini, Mauro Tognon

**Affiliations:** ^1^Department of Translational Medicine and for Romagna, University of Ferrara, 44121 Ferrara, Italy; ^2^Department of Chemical, Pharmaceutical and Agricultural Sciences-DOCPAS, University of Ferrara, 44121 Ferrara, Italy; ^3^Department of Medical Sciences, Section of Experimental Medicine, School of Medicine, University of Ferrara, 44121 Ferrara, Italy; ^4^Department and Biomedical Sciences and Specialized Surgeries, Section of Neurology, University of Ferrara, 44121 Ferrara, Italy; ^5^Interdepartmental Research Center for the Study of Multiple Sclerosis and Inflammatory and Degenerative Diseases of the Nervous System, University of Ferrara, Ferrara, Italy; ^6^Laboratory for Technologies of Advanced Therapies, Department of Medical Sciences, University of Ferrara, 44121 Ferrara, Italy

## Abstract

Multiple sclerosis (MS) is a neurological disease characterized by immune dysregulations. Different viruses may act as MS triggering agents. MS patients respond differently to distinct viruses. The aim of our study is to verify the association between the polyomavirus BKPyV and MS, together with other neurological diseases, through the investigation of serum IgG antibodies against the virus. Sera were from patients affected by MS and other neurologic diseases, both inflammatory (OIND) and noninflammatory (NIND). Control sera were from healthy subjects (HS). Samples were analyzed for IgG antibodies against BKPyV with an indirect ELISA with synthetic peptides mimicking the viral capsid protein 1 (VP1) antigens. As control, ELISAs were carried out to verify the immune response against the Epstein-Barr virus (EBV) of patients and controls. In addition, we assessed values for total IgG in each experimental groups. A significant lower prevalence of IgG antibodies against BKPyV VP 1 epitopes, together with a low titer, was detected in sera from MS patients and other inflammatory neurologic diseases than HS. In MS patients and OIND and NIND groups, the EBV-antibody values and total IgG did not differ from HS. Experimental data indicate that patients affected by neurological diseases, including MS, are poor responders to BKPyV VP 1 antigens, thus suggesting specific immunologic dysfunctions for this polyomavirus. Our findings are relevant in understanding the immune reactions implicated in neurological disorders.

## 1. Introduction

It has been reported that multiple sclerosis (MS), which is a disease of the central nervous system (CNS), arises in the context of innate immune defects of predisposed patients, i.e., the genetic background of the host [1]. The mechanism that triggers this autoimmune disorder remains to be fully elucidated. Many investigations reported that different viral agents, in association with patient genetic factors, may play a role in the MS onset/progression [2]. Data obtained in animal models of virus inducing demyelination, laboratory findings, and epidemiological results, altogether demonstrated that viruses and host genetic factors can interact to cause immune-mediated demyelination [3]. It has been suggested that ubiquitous viruses may act as environmental “triggers” involved in the MS disease process [4]. There is currently no consensus on the cause of MS, whereas different hypotheses vary from idiopathic loss of self-tolerance to molecular mimicry to persistent virus infections. It is generally accepted that MS involves a combination of genetic predisposing factors and environmental influences [5]. Major predisposing factors for MS are certain tissue antigen types, such as HLA DRB1∗15 : 01, vitamin D deficiency, smoking, obesity, and infection with Epstein-Barr virus (EBV). EBV infection appears to be a necessary, but not sufficient requirement for developing MS [6]. How EBV may influence MS risk remains unknown.

The human polyomavirus (HPyV) BK (BKPyV) infects and multiplies in different tissues including the central nervous system (CNS). BKPyV, which was isolated for the first time in a kidney transplant recipient [7], naturally infecting humans. This HPyV is considered ubiquitous in different populations worldwide. After the primary infection, which usually occurs in early childhood, BKPyV remains lifelong, clinically unapparent, in the immunocompetent host. BKPyV, during the persistent phase of infection in healthy hosts, multiplies with a low titer generating a limited viruria in a fraction of subjects [8]. Many cell types, including urogenital cells, peripheral blood mononuclear cells, and CNS cells, seem to be involved in the BKPyV dissemination and persistence [9].

BKPyV is ubiquitous in humans, with an estimated seroprevalence in adults of 75% (range 46%–94%) [10]. The different populations analyzed in previous studies may account for the distinct BKPyV prevalence. Recent studies reported a lower BKPyV seroprevalence [11–13]. These data were obtained with specific indirect ELISAs, with synthetic peptides, which do not cross-react with the other homologous polyomaviruses, such as JCPyV and SV40.

The early age of seroconversion, along with the detection of the virus in tonsillar tissue, suggest that the respiratory way is the BKPyV portal of entry [14]. The virus is reactivated in immunosuppressed patients, particularly in those with cellular immune deficiency, such as organ transplant recipients, allowing its detection in urine and blood [15]. In immune-suppressed patients, BKPyV may reactivate causing severe diseases, such as nephropathies and hemorrhagic cystitis, which were reported in the kidney and bone marrow transplant patients [16]. Moreover, in severe immunosuppressed hosts, together with other predisposing factors, BKPyV may induce meningoencephalitis [17].

The aim of this study was to verify the association of BKPyV with neurological diseases, including MS. To this purpose, serum samples from patients affected by neurologic diseases were analysed using indirect ELISAs with BKPyV VP1 mimotopes, as synthetic peptides. As control, total IgG antibody profile and the EBV antibody response were investigated.

## 2. Materials and Methods

### 2.1. Sera

Sera (*n* = 570) from neurologic disease- (ND-) affected patients (*n* = 324) were collected at the Neurological Clinic/Multiple Sclerosis (MS) Centre, Section of Neurology, School of Medicine, University of Ferrara, whereas samples from normal healthy subjects (HS; *n* = 246) were obtained from the clinical laboratory analysis, University Hospital of Ferrara, Ferrara, Italy. MS and non-MS patients have been admitted during the last 10 years. Sera from ND patients were affected by (i) MS, (ii) other inflammatory neurologic disorders (OIND), and (iii) noninflammatory neurologic diseases (NIND). None of the study patients underwent treatment with immune-suppressants or immune-modulating drugs, including steroids, at the time of sampling. Sera from HS were collected in two cohorts with a different mean age and different percentage of male: HS1 similar to MS cohort and HS2 similar to the OIND and NIND cohorts. The characteristics of ND-affected patients and HS are reported in Table 1. Anonymously collected sera were coded with indications of age, gender, and pathology. All subjects/patients gave written informed consent in accordance with the Declaration of Helsinki. The Ethical Committee (EC) of the Province of Ferrara, Italy, approved the study, protocol number 151078.

### 2.2. Indirect ELISA

The immunological ELISAs, to detect serum IgG antibodies (Abs) against BKPyV, were employed as reported before [13]. Briefly, immunological plates were coated with two BKPyV VP1 peptides corresponding to viral antigens (Ags). The two mimotopes, named BKPyV L and M peptides, were from the viral protein 1 (VP1: 669-689 aa). Computer-assisted analyses allowed us to select the specific BKPyV peptides from the late viral region by comparing the VPs, with the amino acids of the closely related polyomaviruses JC (JCPyV) and simian virus 40 (SV40), as well as with other less homologue polyomaviruses.

The two mimotopes a.a. sequences were BKPyV Peptide L: NH2-LKLSAENDFSSDSPERK-COOH and BKPyV Peptide M: NH2-MLNLHAGSQKVHEHGGGK-COOH.

Mimotops VP1 L and VP1 M amino acid sequences were studied in relation on their stable secondary structure formation. PSIPRED server was employed to perform the analysis [18]. Peptide sequences were mapped on native virion proteins to verify structural similarities. A computational prediction carried out by I-TASSER server was used to obtain monomeric forms of VP1-L and VP1-M [19]. Molecular visualizations were performed by PyMOL (PyMOL Molecular Graphics System, version 1.3, Schrödinger, LLC). Computational tools were available through ExPASy server [20].

The two peptides were synthesized and purchased from UFPeptides, s.r.l., Ferrara. Italy. The immune response, against the two BKPyV peptides L and M, has been reported before by our groups [11–13]. Earlier indirect ELISA investigations showed that the two BKPyV VP1 mimotopes did not cross-react with SV40 and JCPyV hyperimmune sera used as controls [13].

The different phases of the assay were as follows: (i) peptide coating, (ii) peptide blocking, (iii) primary antibody adding, (iv) secondary antibody adding, (v) dye treatment, (vi) spectrophotometric reading, and (vii) cut-off determination [13].

- *Peptide coating:* ninety-six well flat-bottom wells (Nunc-immuno plate Poly- Sorp, CelBio, Milan, Italy) were coated with 5 *μ*g of the selected peptide (BKPyV VP1 L and BKPyV VP1 M) for each well, which were diluted in 100 *μ*L of coating buffer (Candor Bioscience, Weissensberg, Germany). The plates were left at 4°C for 16 hours to allow the peptide to completely cover the bottom well. To eliminate uncoated peptide the plates were rinsed with the washing buffer three times (Candor Bioscience, Weissensberg, Germany). Peptides were synthesized with standard procedures and purchased from UFPeptides s.r.l., Ferrara, Italy
- *Blocking phase* was made with 200 *μ*L/well of the blocking solution (Candor Bioscience, Weissensberg, Germany) at 37°C for 90 minutes. To remove the blocking solution, washing buffer was used to rinse the plates three times
- *Primary antibody*: serum samples were diluted in a low cross-buffer at a 1 : 20 in a final volume of 100 *μ*L (Candor Bioscience, Weissensberg, Germany) and then added to 96 plastic wells. A human negative peptide, the neuropeptide S (hNPS) with the a.a. sequence SFRNGVGTGMKKTSFQRAKS, was employed as a control. This human neuropeptide is nonliked to BKPyV [11, 21]
- *Secondary antibody*: after 90 minutes of incubation, a new triple rinsing cycle was repeated as described above. Then, in each well, the secondary human antibody solution was added. Secondary antibody is a goat antihuman or antirabbit immunoglobulin G (IgG) heavy (H) and light (L) chain–specific peroxidase conjugate (Calbiochem-Merck, Darmstadt, Germany) diluted 1: 10,000 in 200 *μ*L of low cross-buffer (Candor Bioscience, Weissensberg, Germany). For 90 minutes, the reaction mixture was incubated at room temperature
- *Dye treatment*: after incubation, the plates were rinsed three times with the washer buffer and then treated with 100 *μ*L of 2,2-azino-bis 3-ethylbenzthiazoline-6-sulfonic acid solution; ABTS (Sigma–Aldrich, Milan, Italy). The colorimetric process was stopped after 45 minutes with 100 *μ*L of 0.1 M citric acid
- *Spectrophotometric reading* at a wavelength (*λ*) of 405 nm (Thermo Electron Corp., model Multiskan EX, Finland). The cut-off was determined as reported. The apparatus “Wellwash 4 Mk 2” (Thermo Electron Corp, Vantaa, Finland) was used in ELISA to remove the solutions/sera and to rinse the plates. The Wellwash 4 Mk 2 is a semiautomatic microplate washer for 96-well plate, which includes pump and washer units
- *Cut-off determination*: in each test, the cut-off was set by adding the OD mean readings of the three negative control sera three times (+3 SD) to the standard deviation (SD) [21]. Sera containing antibodies against BKPyV were considered VP1-positive when they reacted to both late region peptides and when indirect ELISA testing produced the same positive findings three times. A similar technical approach, with different mimotops, was employed in previous studies to detect serum IgG Abs against JCPyV [22]

### 2.3. Virus, Cells, Hemagglutination (HA), and Hemagglutination Inhibition (HAI) Assays

BKPyV, Gardner strain, genotype Ia (ACCESSION NUMBER: V01108.1), kindly supplied by Dr. S.D. Gardner, was employed as the viral working stock. BKPyV was grown in Vero cells, as previous described [7, 13]. Hemagglutination (HA) and hemagglutination inhibition (HAI) assays were carried out as described in detail elsewhere [13]. The comparison between the indirect ELISA and HAI tests is of fundamental significance. Indeed, HAI assay is highly specific because only BKPyV-positive sera can inhibit the capability of BKPyV virions to agglutinate human erythrocytes, group 0, Rh^+^. Human sera, analyzed using the hemagglutination inhibition (HAI) assay, were serially diluted from 1 : 16; 1 : 32; 1 : 64; to 1 : 128. HAI assay evaluates both the presence and titer of antibodies against BKPyV. This assay was carried out as a control to verify the data obtained with the new indirect ELISA. To this end, 150 randomly selected sera out of 570 samples assayed by indirect ELISA were tested by HAI assay.

### 2.4. Total IgG Values

Total IgG concentrations in serum samples of MS patients (*n* = 20), NIND (*n* = 20), and OIND patients (*n* = 20) and healthy subjects (*n* = 20) were assessed using the commercial kit “Human IgG ELISA Kit” according to the manufacturer's instructions (catalog number RAB0001 Millipore, Milan, Italy). The serum samples analyzed for total sera IgG variability were chosen from all samples, in an equal number of sera for the four different cohorts analyzed (HS, MS, OIND, and NIND) in order to be representative of the individual cohort. In addition, the samples within the same court were chosen randomly. The ELISA plate was read spectrophotometrically (Thermo Electron Corp., model Multiskan EX, Finland) at a wavelength of 450 nm. The lower threshold for detection of IgG with this method is 20 pg/mL. The reference intervals for healthy adults was IgG 700–1,600 mg/dL [23, 24].

### 2.5. IgG against EBV-VCA

The samples analyzed for IgG against Epstein-Barr viral capsid antigen (VCA) antibodies variability were chosen among all samples, in an equal number of sera for the four different cohorts analyzed (HS, MS, OIND, and NIND) in order to be representative of the individual cohort. In addition, the samples within the same court were chosen randomly. EBV-VCA antibodies were measured in diluted sera by a quantitative IgG enzyme-linked immunosorbent assay (ELISA) kits (IBL International, Hamburg, Germany, catalog number RE56281) following the manufacturer's instruction. The IgG antibody titer was quantified by drawing a curve with the four included standards (1, 10, 50, and 200 EBV units (U)/mL) and expressed as U/mL. EBV-VCA Ab datasheet defines U (units) as the quantity of antibodies against EBV-VCA, which results in an OV value of 0.004. For the quantification, the cut-off index (COI) of the samples can be obtained as follows:

COI = OD (sample)/OD Standard B.

Samples with COI < 0.8 are considered negative; samples with COI 0.8-1.2 are considered equivocal; samples with COI > 1.2 are considered positive.

### 2.6. Statistical Analyses

Chi-square test was used to compare the prevalence of BKPyV antibodies in different groups. Statistical analyses were carried out using GraphPad Prism version 6.0 for Windows (GraphPad, La Jolla, CA, USA). *p* values < 0.05 were considered statistically significant. The prevalence of EBV-VCA-positive serum samples from MS patients was compared with the prevalence detected in healthy individuals and other cohorts. All data are expressed as percentages. To determine the significance between 2 groups, Fisher's exact test was used. The total IgG and EBV-VCA antibodies titers' value were statistically analyzed using ANOVA and Bonferroni's multiple comparisons test. All computational analyses were performed with Prism 6.0 (GraphPad software, San Diego, CA). For all tests, *p* was considered to be statistically significant when *p* < 0.05.

## 3. Results

### 3.1. Detection of IgG Abs against BKPyV

**Figure 1:**
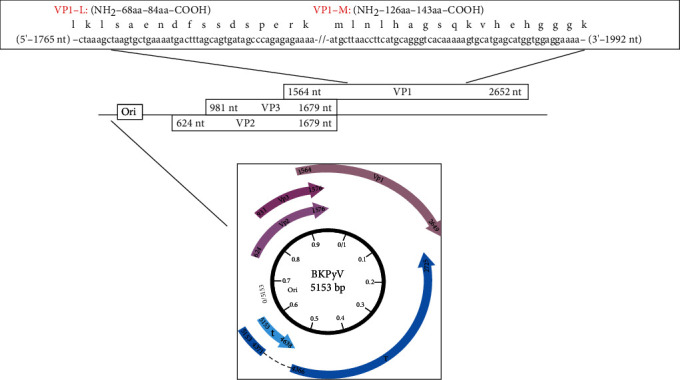
Mimotopes' sequences. BKPyV genome and the two selected peptides from the late region employed in indirect ELISA. The circle at the bottom represents the BKPyV genome, with the map unit from 0 to 100 running in a clockwise direction (inner circle, black). Ori is the origin of viral DNA replication, which is composed of 5,153 nucleotides, nt (0/5153). BKPyV nucleotide sequence is reported in http://www.ncbi.nlm.nih.gov/genome. BKPyV early and late genes are transcribed in both anticlockwise and clockwise directions, respectively (gray arrows); numbers indicate nt. The large T antigen (T) and small t antigen (t) are encoded by the early region (T antigen, exon 1, 5,153–4,371 nt, intron 1, 4,372–4,365 nt, and exon 2, 4,366–2,725 nt; t ag 5,153–4,638 nt). Viral capsid proteins (VP) 1–3 are codified by the late region (VP1, 1,564–2,652 nt; VP2, 624–1,679 nt; VP3, 981–1,679 nt). The late coding region is expanded in the upper part of the figure. The selected peptides, namely, VP1 L and VP1 M, are indicated in the schematic representation of the late region, which encodes for the VP 1–3 capsid proteins. VP1 L a.a. 68–84 (17 aa) and VP1 M aa 126–143 (18 aa), respectively, together with the nt sequences.

To investigate the BKPyV infection in sera from MS, OIND, and NIND patients and HS, indirect ELISAs were employed to analyze IgG Abs reacting to BKPyV VP1 L and M mimotopes (Figure 1). Together with BKV VP 1 mimotopes [13, 25], a nonspecific human peptide hNSP [25] was employed as a negative control. Immunological results allowed us to assess the presence of serum IgG antibodies against BKPyV in MS, OIND, and NIND patients. The control was represented by sera from two different cohorts of healthy subjects, HS1 and HS2, selected to match the median age and gender ratio of the different cohorts of patients under analyses. Specifically, HS1 matches the MS group, whereas HS2 matches the OIND and NIND groups (Table 1). Sera under investigation were employed diluted at 1 : 20 for reactivity. BKPyV-positive sera were those reacting to both VP1 L and M peptides. Serum IgG antibodies against VP1 L and M peptides reacted to these mimotopes with a similar prevalence. The difference in term of positive/negative results for VP1 L and VP1 M are not statistically significant (*p* > 0.05) (Table 2).

BKPyV Abs were detected in 22% MS, 21% OIND, 30% NIND, 70% HS1, and 66% HS2 (Table 2). The prevalence of BKPyV antibodies in MS patients is statistically significant lower that HS1 (*p* < 0.0001). The different prevalence revealed in MS, OIND, and NIND patients was not significant (*p* > 0.05). BKPyV Abs prevalence in OIND and NIND patients are statistically significant lower that those detected in HS2 (*p* < 0.0001). The statistically significant difference between OIND and HS2 does not change with or without PML cases. However, our hospital records indicate that these two PML cases were not associated with JCPyV. *χ*^2^ test was employed for the statistical analysis. In ELISAs, the control human peptide hNPS [25] did not react to Abs against BKPyV present in positive sera. Indeed, the low OD values (0.088-0.098) are similar to those of BKPyV-negative sera [13]. The prevalence of BKPyV-positive sera was 70% and 66%, in HS1 and HS2, respectively.

**Figure 2:**
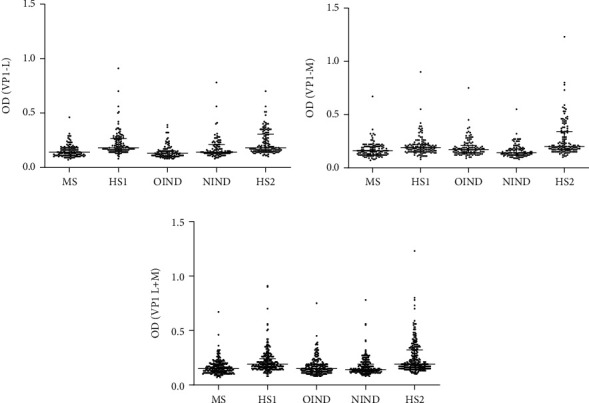
Serologic profile. Serologic profile of serum antibody reactivity (OD) to BKPyV mimotopes VP1 L (a) and VP1 M (b) and VPs L+M (c). (a) In ELISAs with VP 1 L peptide, the mean OD of sera (mean ± SEM) in MS (0.15 ± 0.06) were statistically significant lower (^∗∗∗^*p* < 0.0001) than that in HS1 (0.22 ± 0.11). Moreover, the mean OD of sera in OIND and NIND (0.14 ± 0.06 and 0.18 ± 0.10, respectively) were statistically significant lower (^∗∗∗^*p* < 0.0001, *p* = 0.0003, respectively) than that in and HS2 (0.23 ± 0.11). The mean OD of sera in MS, NIND, and OIND are similar; the mean OD of sera in HS1 and HS2 are not statistically different, too. (b) In ELISAs with VP 1 M peptide, the mean OD of sera (mean ± SEM) in MS (0.17 ± 0.07) were not significantly lower (*p* > 0.05) than HS1 (0.21 ± 0.10). Instead, the mean OD of sera in OIND and NIND (0.19 ± 0.09 and 0.16 ± 0.06, respectively) were statistically significant lower (^∗∗∗^*p* < 0.0001) than HS2 (0.27 ± 0.17). The mean OD of sera in MS, NIND, and OIND are similar; however, the mean OD of sera in HS1 and HS2, probably due to the different ages, is statistically different (^∗∗∗^*p* < 0.0001). (c) Adding the mean OD of sera obtained in ELISAs with VP 1 L peptide and with peptide VP 1 M, (mean + SEM) in MS (0.16±0.07) were statistically significant lower (^∗∗∗^*p* < 0.0001) than HS1 (0.21 ± 0.11). Moreover, the mean OD of sera in OIND and NIND (0.17 ± 0.08 and 0.17 ± 0.08, respectively) were statistically significant lower (^∗∗∗^*p* < 0.0001) than HS2 (0.25 ± 0.14). The mean OD of sera in MS, NIND, and OIND are similar; however, the mean OD of sera in HS1 and HS2, probably due to the different ages, are statistically different (^∗∗^*p* < 0.01). Statistical analysis was performed using ANOVA and Newman-Keuls comparison test.

Serologic profiles of serum antibody reactivity show the OD, obtained with mimotope VP1 L (Figure 2(a)), mimotope VP1 M (Figure 2(b)), and the two mimotopes together (Figure 2(c)) reflect the titer of the antibodies against BKPyV present in the single sample.

ODs of sera from MS, OIND, and NIND were lower than OD of HS1 and HS2, with most of the differences statistically significant. MS vs. HS1 was statistically significant for mimotope VP1 L and for the sum of the 2 mimotopes employed. OIND vs. HS2 and NIND vs. HS2 were always statistically significant. OD profiles of serum samples from MS, NIND, and OIND had similar values. However, the mean OD of sera in HS1 and HS2 is statistically different for the VP 1 M mimotope and VP1 L+M peptides.

### 3.2. Detection of BKPyV Antibodies by HAI Assay

In HAI experiments, as done in ELISAs, two HS cohorts were selected to match the median age and the gender ratio of patients. HS1 matches with the MS group, whereas HS2 matches the OIND and NIND groups. Diluted sera, 1 : 16, 1 : 32, 1 : 64, to 1 : 128, were assayed by HAI test to verify both the presence of Abs against BKPyV and their titer. Data obtained with sera at low dilutions, 1 : 32 or 1 : 64, which may carry BKPyV Abs at high concentration and sera at high dilutions with Abs at low concentration, were not taken into consideration because they may give to false positive reactions and negative results, respectively. Indeed data from the literature and previous results from our group indicate that the dilution 1 : 128 represents the right concentration of BKPyV Abs to test the inhibitory effect on BKPyV virions [26, 27]. Hemagglutination inhibition (HAI) test indicates that the prevalence of BKPyV antibodies, at the serum dilution 1 : 128, was 13%, 17%, 27%, 63%, and 57%, for MS, OIND, NIND, HS1, and HS2, respectively (Table 3).

The prevalence of BKPyV antibodies in MS patients is statistically significant lower that HS1 (^∗∗^*p* < 0.001). Moreover, the BKPyV Abs prevalence in sera from OIND and NIND patients is statistically significant lower than HS2 (*p* < 0.05). The prevalence of BKPyV antibodies detected in sera from MS, OIND, and NIND patients did not differ statistically (*p* > 0.05) (Table 3).

Interestingly, the prevalence of BKPyV-positive samples by HAI did not differ from that determined by the innovative indirect ELISA with L and M mimotopes (*p* > 0.05) (Table 2). Moreover, the indirect ELISA and HAI assays showed good rates of sensibility and specificity, which were 96% (145/150) and 93% (140/150), respectively.

### 3.3. Total Serum IgG

**Figure 3:**
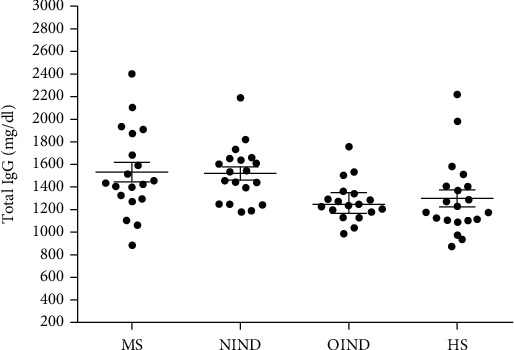
Total IgG variability in sera from MS, NIND, OIND, and HS. Data are graphed as scatter dot plot where IgG mean values and their standard error mean (SEM) are marked by short horizontal lines. The IgG levels were not statistically different among the cohorts (*p* > 0.05).

The total immunoglobulin G (IgG) concentrations showed normal distribution in all patients and healthy subjects (Figure 3, Table 4). The IgG levels (IgG mean value ± SEM; mg/dL) present in serum samples of all MS patients are 1,532 mg/dL ± 86.96 mg/dL, in OIND patients are 1,275 mg/dL ± 43.17 mg/dL, in NIND patients are 1,520 mg/dL ± 58.08 mg/dL, and in HS are 1,299 mg/dL ± 74.60 mg/dL. All these IgG values are among normal concentrations, as reported for HS [23, 24]. Total IgG values detected patients were not statistically different from HS (*p* > 0.05), by ANOVA and Bonferroni multiple comparisons test.

### 3.4. IgG against EBV-VCA

**Figure 4:**
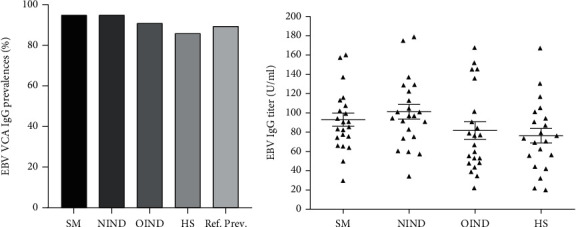
EBV seroprevalence and titer in sera from MS and other neurological diseases affected patients. (a) Prevalence of immunoglobulin G (IgG) antibodies reacting to EBV-VCA in MS and other neurological diseases affected patients from our data and prevalence, as reported by Wong and colleagues [28]. (b) The titer values of EBV-VCA are shown as scatter dot plot, where IgG mean values and their standard error mean (SEM) are marked by short horizontal lines. The VCA-EBV IgG levels were not statistically different among the cohorts (*p* > 0.05).

Sera from MS, OIND, NIND, and healthy subjects were tested by indirect ELISAs for IgG Ab reactive to EBV-VCA (Figure 4, Table 4). EBV-VCA was positive in 95% of MS patients, in 95% of NIND, in 91% of OIND patients, and 86% of healthy individuals, without any significant difference among the groups (*p* > 0.05). The value of IgG against EBV-VCA were measured in MS patients and other cohorts. The EBV-VCA IgG levels (mean value ± SEM, U/mL) present in serum samples of MS patients are 93 ± 6.8 U/mL, in NIND patients are 101 U/mL ± 7.6 U/mL, in OIND patients are 82 U/mL ± 9.1 U/mL, and in HS are 76 U/mL ± 7.6 U/m. The EBV-VCA IgG values detected in patients and other cohorts were not statistically different (*p* > 0.05).

## 4. Discussion

In our investigation, sera from patients affected by different neurological diseases, including MS, OIND, NIND, and healthy individuals, were analyzed for anti-BKPyV IgG antibodies. The prevalence of Abs against BKPyV VP1 in patients affected by MS, OIND, and NIND was lower compared to the two different cohorts HS1 and HS2, which are the corresponding controls (Table 2).

Data obtained by HAI, which is a high specific assay, confirmed ELISA results with BKPyV VP1 mimotopes. Indeed, MS, OIND, and NIND sera have a lower prevalence of antibodies against BKPyV, than HS1 and HS2 (Table 3). A similar prevalence was detected in other cohorts of HS published before [13] but are different from data reported by other investigators [10, 29]. Specifically, Egli and Knowels revealed a BKPyV prevalence in HS higher than our cohorts of healthy individuals. The different prevalence could be due to the distinct immunological methods employed or different populations analyzed.

MS patients included in this study, at the time of the serum collection, were not subjected to any immunomodulatory therapy. Accordingly, the low prevalence of BKPyV Abs detected in MS sera cannot be ascribed to the immune-modulatory therapy.

In our experiment, SV40-positive and JCPyV-positive sera used as control did not react with BKPyV VP1 L and M mimotopes. Therefore, our data confirm that BKPyV VP 1 mimotopes used herein are specific and did not cross react with SV40 and JCPyV, as reported before with other human serum samples [13].

Ubiquitous human viruses could act as environmental risk factors, acting with other factors, in the MS onset. At present, it is not known if a single virus or different viruses are associated with MS. BKPyV is a able to infect and replicate in many different tissues, including tissues of central (CNS) and peripheric nervous systems (PNS). Its DNA sequences were revealed in CSF of patients with suspected meningitis or encephalitis [30] and in brain tumors of different histotypes [31]. BKPyV-DNA was also detected in an encephalitis case of immunosuppressed patient [32]. In addition, normal brain tissues tested BKPyV-positive [9]. Moreover, in two distinct studies BKPyV sequences were revealed with a prevalence of 59.5% and 22.2%, respectively, in urine samples from MS patients treated with the mab natalizumab [33, 34]. These data confirm that BKPyV may reactivate both in immunocompetent subjects and immunosuppressed patients.

Our investigation reports for the first time a significant low prevalence of BKPyV Abs in sera from patients affected by neurologic diseases, including MS. We may speculate that these patients, because affected by specific immune impairments, are poor responders to BKPyV antigens (Ags). Their immune-dysfunctions may allow BKPyV to escape the immune surveillance, thus exerting its pathological effect in CNS. A similar effect has been reported for the human herpetic virus HHV-6 [35]. The authors suggested that the mechanism of immune escape of HHV-6 in autoimmune disease may occur with the concomitant abnormal expression of histocompatibility molecules, thus favoring the presentation of autoantigens [35]. Many investigations focused on the research of antibodies against different viruses in blood or other body fluids of MS patients to study the possible association between MS and different viruses. This issue is controversial, as some studies reported high Abs against certain viruses in cases vs. control, while other investigators detected low Abs against the same viruses. A recent investigation reported that IgG levels in MS patients and HS against distinct herpesviruses do not differ. Higher IgG prevalence against EBV was detected in some studies [36–38] whereas Wagner et al. reported a lower prevalence and titer of anti-EBV nuclear antigen 1 (EBNA 1) IgG antibodies in MS sera, indicating that these patients are deficient in controlling the EBV infection/reactivation [39]. Indeed, there is a considerable body of epidemiological evidence on the association between EBV and MS. In our study, EBV-VCA IgG in MS patients did not differ in healthy subjects and other controls groups represented by NIND and OIND patients. In agreement, the titer of anti-EBV capsid antigen IgG had similar levels between patients and controls. Our reported seroprevalence value of EBV-VCA is consistent with the earlier investigations on MS and healthy subjects [23, 40]. In the investigated MS cohort, the correlation of autoimmune disease with EBV infection is probably not evident because the synergy between infection and other factors is needed to trigger MS [23, 41]. In our study, we revealed a different prevalence of IgG against BKPyV in patients and healthy subjects by indirect ELISAs. In this study, sera from healthy subjects reacted to BKPyV VP1 mimotopes with a similar prevalence reported by other studies. The small difference in prevalence reported by different studies is not significant.

Of note, BKPyV VP1 antibodies detected with our innovative ELISAs, with a prevalence of 66%-70% depending on the cohorts investigated, reported herein and in our previous investigations, is in agreement with the literature of the field [12, 29, 42].

Furthermore, early immunological assays, which employed as a viral antigen the recombinant VP1 or VLPs, always showed cross-reactivity among three highly homologue polyomaviruses, BKPyV, JCPyV, and SV40, thus affecting the specificity of immunological data. In earlier studies, different authors developed enzyme immunoassays (EIAs) using virus-like particles (VLPs) produced in the baculovirus expression system [43, 44]. The cross-reactivity among these polyomaviruses is due to their high homology, which is approximately 70% [44, 45]. Our indirect ELISA is specific in discriminating BKPyV VP1-negative from BKPyV VP1-positive subjects/patients. In addition, there is not cross-reactivity among the three high homologue polyomaviruses. Our innovative indirect ELISA with BKPyV VP1 mimotopes is a new useful method to detect specific IgG antibodies against this virus in human sera without cross-reactivity with the closely related JCPyV and SV40.

This investigation was addressed to verify the association of neurological diseases, including MS, with BKPyV. To this purpose, sera were tested for BKPyV antibodies in indirect ELISAs using BKPyV VP1 synthetic peptides, as mimotopes/antigens [11–13, 21]. Overall, data obtained with the ELISA test for the determination of antibodies against BKPyV show a lower prevalence and a lower antibody titer in sera of neurological disease patients compared to healthy subjects [11–13, 21]. There is no previous scientific report on this issue. We may speculate that the low BKPyV seroprevalence detected in neurological diseases patients could be due to an impaired immune response against this human polyomavirus or alternatively to a reduction of BKPyV infection.

## 5. Conclusions

Our immunological investigations indicate specific dysregulations in IgG Abs against BKPyV VP1 in sera from MS, OIND, and NIND patients. Serologic profile of serum antibody reactivity (Figure 2) shows that the titer of antibody against BKPyV VP1 mimotopes in MS, OIND, and NIND sera were lower than HS1 and HS2. The alterations leading to the immunological defect is not known. It is possible that different cases may account for this immunological profile, including the chronic inflammation of MS and OIND patients could be responsible of several immune impairments as reported before [46].

This is the first investigation reporting on the low prevalence of IgG antibodies against BKPyV in sera from ND patients, including MS. Our data did not allow to verify if BKPyV plays a role in the MS onset. Additional experiments, such as the evaluation of the viral DNA load, virus isolation and genotyping, HLA characterization of the host are needed. These analyses are feasible, and they will be part of next investigations.

## Figures and Tables

**Table 1:** Characteristics of MS, OIND, and NIND patients and healthy subjects (HS).

Serum (number)	Male (%)	Mean yrs ± SD (range)	Subtype	Number of subtype	BKPyV-positive sample/sample analyzed (%)	
MS (118)	36	3811.2 (13-68)	RR	103	21/103	(20)
			SP	10	3/10	(30)
			PP	5	2/5	(40)

OIND (106)	49	5415.5 (18-88)	Inflamm. Demyelinating	35	6/35	(17)
			Mening./Enceph./myelitis	44	12/44	(27)
			Mononeuritis	5	1/5	(20)
			Comnnectivitis/vasculitis	13	3/13	(23)
			PML	2	0/2	—
			Polyneuropathy	7	0/7	—

NIND (100)	46	5316.8 (21-85)	ALS	12	3/12	(25)
			Dementia	9	1/9	(11)
			MSA	2	0/2	—
			AVM	4	0/4	—
			Migraine	11	7/11	(64)
			Toxic encephalopathy	2	0/2	—
			Epilepsy	7	2/7	—
			Hereditary ataxia	3	1/3	(33)
			Brain tumor	8	0/8	—
			Hydrocephalus	2	0/2	—
			Spondylotic myelopathy	4	1/4	(25)
			Peripheral neuropathy	8	2/8	(25)
			Pseudotumor cerebri	4	0/4	—
			Funicular myelopathy	1	1/1	(100)
			Stroke/TIA	23	12/23	(52)

HS-1 (121)	32	3910 (17-63)	Healthy subjects	121	85/121	(70)

HS-2 (125)	42	5317 (17-89)	Healthy subjects	125	83/125	(66)

MS: multiple sclerosis; OIND: other inflammatory neurological disease; NIND: noninflammatory neurological disease; HS: healthy subjects; RR: relapsing-remitting; SP: secondary-progressive; PP: primary-progressive; PML: progressive multifocal leucoencephalopathy; ALS: amyotrophic lateral sclerosis; MSA: multiple system atrophy; AVM: arteriovenous malformation; TIA: transient ischemic attack.

**Table 2:** Prevalence of immunoglobulin G antibodies reacting to BKPyV viral protein 1 (VP1) mimotopes L and M.

Serum type	Number of patient/subject	Male (%)	Median age (yrs)	Number of positive sample (%)		
				VP 1 L	VP 1 M	VP (L+M)
MS	118	36	38	30 (25)	35 (30)	26 (22)
HS-1	121	32	39	91 (75)	92 (76)	85 (70)
OIND	106	49	54	23 (22)	33 (31)	22 (21)
NIND	100	46	53	35 (35)	33 (33)	30 (30)
HS-2	125	42	53	83 (66)	88 (70)	83 (66)

Human sera tested positive for the single peptide VP 1 L or VP 1 M are reported in columns 5 and 6, respectively, whereas positive sera for both peptides L+M are in column 7. Sera were from patients affected by MS, OIND, and NIND and from healthy subjects, HS-1 and HS-2.

**Table 3:** Prevalence of serum IgG antibodies against BKPyV analyzed by hemagglutination inhibition (HAI) assay.

Serum	Number of subject	Median age ± SD	Male (%)	Number of positive samples (%) at the dilution			
				1 : 16	1 : 32	1 : 64	1 : 128
MS	30	39 ± 12	27	10 (33)	8 (27)	7 (23)	4 (13)^∗∗^
HS-1	30	38 ± 5	27	24 (80)	22 (73)	19 (63)	19 (63)
OIND	30	50 ± 12	60	12 (40)	7 (23)	7 (23)	5 (17)°
NIND	30	52 ± 17	57	10 (33)	12 (40)	8 (27)	8 (27) °
HS-2	30	53 ± 3	60	24 (80)	22 (73)	20 (67)	17 (57)

The prevalence of BKPyV antibodies in MS patients is significantly lower than in HS1 ^∗∗^*p* < 0.001, whereas no significant was the different prevalence detected in MS vs. OIND and vs. NIND patients (*p* > 0.05). Moreover, the prevalence of BKPyV antibodies in OIND and NIND patients are significantly lower than HS2 (*°p* < 0.05, respectively). Statistical analysis was performed using the *χ*^2^ test.

**Table 4:** EBV-VCA IgG prevalence and total IgG antibodies detected in serum samples from MS patients, NIND, and OIND compared to HS.

	EBV-VCA IgG positive sample/total (prevalence)	EBV-VCA IgG positive value ± SEM (U/mL)	Total IgG Mean value ± SEM (mg/dL)
SM	21/22 (95%)	93 ± 6.8	1,532 ± 87
NIND	21/22 (95%)	101 ± 7.6	1,520 ± 58
OIND	20/22 (91%)	82 ± 9.1	1,275 ± 43
HS	19/22 (86%)	76 ± 7.6	1,299 ± 75

IgG antibodies against EBV (prevalences and quantification) detected in serum samples from MS, NIND, and OIND patients and healthy subjects (HS). Total IgG antibodies were detected in each serum sample.

## Data Availability

The data supporting the results are available from the corresponding author on reasonable request.
